# Prevalence and factors associated with suicidal ideation among undergraduate nursing students: a cross-sectional study

**DOI:** 10.1186/s12912-026-05259-7

**Published:** 2026-08-18

**Authors:** Amal Ababneh, Fadi Zaben, Mohammad H. Abuadas, Nour Ali Alrida, Mohammad Rabeh

**Affiliations:** 1 Faculty of Nursing, Jerash University, Jerash, Jordan; 2 Department of Pediatric Nursing, Faculty of Nursing, An-Najah National University, West Bank, Palestine; 3 Faculty of Nursing, Yarmouk University, Irbid, Jordan; 4 School of Nursing, Yarmouk University, Irbid, Jordan; 5 Faculty of Pharmacy, Jerash University, Jerash, Jordan

**Keywords:** Suicidal ideation, Nursing students, Prevalence, Mental health, Beck scale for suicidal ideation

## Abstract

**Background:**

Suicidal ideation is a major public health concern and an important indicator of suicide risk among university students. Undergraduate nursing students may be particularly vulnerable because of the combined academic, clinical, and emotional demands of nursing education. Despite growing concern regarding students’ mental health, evidence on the prevalence of suicidal ideation and its associated factors among nursing students in Jordan and Palestine remains limited. This study aimed to determine the prevalence of suicidal ideation and identify factors associated with suicidal ideation among undergraduate nursing students in Jordan and Palestine.

**Methods:**

A cross-sectional study was conducted between October and December 2025 among undergraduate nursing students enrolled in public and private universities in Jordan and Palestine. Participants were recruited using convenience sampling and completed a structured online self-administered questionnaire comprising a researcher-developed sociodemographic questionnaire and the validated Arabic version of the Beck Scale for Suicidal Ideation (BSS). Descriptive statistics were used to summarize participants’ characteristics and estimate the prevalence of suicidal ideation. Spearman’s rank correlation and multiple linear regression analyses were performed to identify factors independently associated with suicidal ideation scores. Statistical significance was established at *p* < 0.05.

**Results:**

A total of 646 undergraduate nursing students participated in the study. Using a BSS cutoff score of ≥ 6, 490 participants (75.9%) were classified as having elevated suicidal ideation. Bivariate analysis demonstrated that previous history of mental illness, economic status, younger age, and sleep duration were significantly correlated with suicidal ideation scores. In the multiple linear regression analysis, previous history of mental illness (β = 0.20, *p* < 0.001), higher perceived economic status (β = 0.13, *p* = 0.001), and younger age (β = −0.10, *p* = 0.010) were independently associated with higher suicidal ideation scores. Sleep duration was not independently associated with suicidal ideation after adjustment for other variables (*p* = 0.059).

**Conclusion:**

Elevated suicidal ideation was highly prevalent among undergraduate nursing students in Jordan and Palestine. Previous history of mental illness, younger age, and higher perceived economic status were independently associated with higher suicidal ideation scores. These findings highlight the importance of implementing routine mental health screening, strengthening accessible psychological support services, and developing culturally appropriate suicide prevention strategies within nursing education programs to promote students’ mental well-being and facilitate early identification of those at increased risk.

**Supplementary Information:**

The online version contains supplementary material available at https://doi.org/10.1186/s12912-026-05259-7.

## Introduction

According to the World Health Organization (WHO), suicide is the third leading cause of death among individuals aged 15–29 years worldwide [1]. Suicide is defined as death resulting from intentional self-inflicted injury, whereas suicidal ideation refers to thoughts of engaging in behaviors intended to end one’s life [1, 2]. Suicidal ideation represents an important precursor to suicide attempts and completed suicide and has become a major public health concern, particularly among young adults [3]. Understanding the prevalence of suicidal ideation and its associated risk factors; defined as demographic, psychological, social, and environmental characteristics that increase an individual’s likelihood of experiencing suicidal thoughts, is essential for developing effective prevention strategies, especially among vulnerable populations.

University students constitute one of the populations at greatest risk for mental health problems because they experience a critical developmental transition characterized by increasing academic responsibilities, greater independence, financial pressures, social adjustment, and uncertainty regarding future careers [4, 5]. These stressors frequently coexist and contribute to psychological distress, including depression, anxiety, sleep disturbances, hopelessness, and suicidal ideation. In the Arab region, these challenges may be further intensified by cultural expectations, limited utilization of mental health services, and stigma surrounding psychological disorders. For example, studies from Lebanon and Jordan have reported high levels of psychological distress among university students, with academic workload, financial difficulties, inadequate social support, and poor sleep quality identified as major contributing factors [5–8]. In Palestine, these academic stressors are compounded by prolonged exposure to political instability, military occupation, movement restrictions, and recurrent violence, all of which adversely affect students’ psychological well-being [9–11]. Recent evidence has demonstrated that Palestinian nursing students experience persistent psychological distress resulting from the combined effects of daily military exposure, educational disruption, financial hardship, and limited access to mental health resources, highlighting the unique challenges faced by this population [12, 13]. Likewise, recent Palestinian evidence has documented substantial academic stress among university students, emphasizing that academic burden is an established challenge within the local educational context [14].

Among university students, nursing students constitute a particularly vulnerable subgroup because they experience the combined demands of rigorous academic education and intensive clinical training. In addition to managing a demanding curriculum, nursing students are required to participate in clinical placements where they are regularly exposed to patient suffering, critical illness, death, ethical dilemmas, and emotionally challenging caregiving situations [15]. The interaction between these clinical experiences and academic pressures may substantially increase psychological distress and contribute to suicidal ideation. Previous studies have consistently shown that nursing students report higher levels of stress, anxiety, depression, and burnout than students in many other academic disciplines [16, 17]. Nevertheless, limited evidence has examined how the interaction between academic workload and clinical training contributes to suicidal ideation, particularly within Middle Eastern settings where sociocultural norms, healthcare systems, and educational environments differ considerably from those in Western countries.

Several individual and contextual factors have been associated with suicidal ideation among university students, including depression, anxiety, previous mental health problems, poor coping strategies, sleep disturbances, social isolation, perceived stigma, and financial difficulties [4, 16, 17]. These factors may be particularly relevant among nursing students because they are simultaneously exposed to demanding academic schedules, emotionally challenging clinical experiences, and concerns regarding future professional responsibilities. In Jordan and Palestine, these challenges occur within similar sociocultural contexts characterized by strong family values, comparable nursing education systems, shared religious beliefs, and persistent stigma surrounding mental illness and suicide, all of which may influence help-seeking behaviors and the recognition of psychological distress [18–20]. Although the two countries share many cultural, educational, and social characteristics, Palestine presents additional contextual stressors related to ongoing political conflict and humanitarian challenges, making comparison across these settings both relevant and informative.

Despite growing recognition of mental health concerns among university students, evidence regarding suicidal ideation among undergraduate nursing students in Jordan and Palestine remains limited. Most previous regional studies have focused on general psychological distress, anxiety, or depression rather than suicidal ideation, and few have investigated the combined influence of sociodemographic, psychological, and educational factors on suicidal thoughts in nursing students. Furthermore, no multicountry study has simultaneously examined suicidal ideation among nursing students in Jordan and Palestine despite their comparable educational systems and shared cultural characteristics. Addressing this gap may provide a broader understanding of the determinants of suicidal ideation and support the development of culturally appropriate prevention strategies.

Therefore, the present study aimed to (1) determine the prevalence of suicidal ideation among undergraduate nursing students in Jordan and Palestine and (2) identify the sociodemographic and psychological factors associated with suicidal ideation in this population. The findings are expected to inform the development of culturally sensitive mental health screening programs and evidence-based interventions that promote psychological well-being and strengthen suicide prevention efforts within nursing education.

## Materials and methods

### Study design

A cross-sectional study was conducted in Jordan and Palestine to assess the prevalence of suicidal ideation and its associated risk factors among undergraduate nursing students. Data were collected between October and December 2025. Undergraduate nursing programs in both countries follow comparable four-year bachelor’s curricula that integrate classroom-based theoretical education with progressively structured clinical training. Clinical placements typically begin during the second academic year and continue throughout the program in diverse healthcare settings, including medical-surgical, pediatric, maternity, psychiatric, and community health services. These academic and clinical requirements expose students to considerable educational and emotional demands that may influence their psychological well-being.

### Participants and sample

Eligible participants were undergraduate nursing students enrolled in bachelor’s nursing programs in Jordan and Palestine, aged 18 years or older, able to access the internet, and willing to provide electronic informed consent. Students enrolled in postgraduate nursing programs (master’s or doctoral level) were excluded because postgraduate education differs substantially from undergraduate education with respect to academic structure, clinical responsibilities, learning outcomes, and psychosocial demands, which could introduce heterogeneity into the study population. Students on academic leave and exchange students were also excluded.

### Sample size

The required sample size was estimated using G*Power version 3.1.9.2 [21]. An a priori power analysis for multiple linear regression (fixed model, R² deviation from zero) was performed assuming a medium effect size (f² = 0.15), statistical power of 80%, a significance level of 0.05, and four predictors included in the final regression model. The analysis indicated that a minimum of 85 participants was required. A total of 646 undergraduate nursing students were recruited, substantially exceeding the required sample size and ensuring adequate statistical power for the planned analyses.

### Data collection

Data were collected using a structured self-administered online questionnaire consisting of two sections. The first section comprised a researcher-developed sociodemographic and academic questionnaire that included age, gender, marital status, religion, nationality, socioeconomic status, academic year, history of mental illness, and sleep pattern. This section was developed specifically for the present study following a review of the relevant literature. Its content validity was evaluated by a panel of five experts in psychiatric nursing, nursing education, and mental health research, who assessed the relevance, clarity, and comprehensiveness of the items. Minor modifications were made based on their recommendations prior to data collection. The English-language version of the researcher-developed questionnaire is provided as Additional File 1. The second section comprised the Arabic version of the Beck Scale for Suicidal Ideation (BSS), validated by Alsalman and Alansari (2019) [22]. The BSS is a previously published 19-item instrument developed by Beck and colleagues to assess the presence and severity of suicidal thoughts during the week preceding assessment [23]. A self-report version of the scale was subsequently introduced in 1988 [24]. Each item is scored on a 3-point ordinal scale ranging from 0 to 2, yielding a total score ranging from 0 to 38, with higher scores indicating greater severity of suicidal ideation. Previous research has demonstrated excellent internal consistency for the BSS, with a Cronbach’s alpha coefficient of 0.93 [24]. A cutoff score of ≥ 6 has been used in previous studies to identify elevated suicidal ideation [25, 26]. In the present study, the BSS demonstrated acceptable internal consistency, with a Cronbach’s alpha coefficient of 0.77.

### Pilot testing

Prior to the main study, the online questionnaire was pilot-tested with 30 undergraduate nursing students who met the study eligibility criteria but were not included in the final analysis. The pilot study aimed to evaluate the clarity, comprehensibility, language, sequence, and estimated completion time of the questionnaire. Minor wording modifications were made based on participants’ feedback. The pilot data were not included in the final analysis.

### Data collection procedure

Data were collected using a secure online questionnaire developed through Google Forms. Following approval from the Institutional Review Board (IRB), the researchers coordinated with deans and faculty members from the nursing faculties of participating universities in Jordan and Palestine to facilitate participant recruitment and distribution of the survey link to eligible nursing students. The survey link was disseminated electronically through university email systems and social media platforms, including WhatsApp, with support from class representatives to maximize outreach and participation. Prior to accessing the questionnaire, participants were presented with an introductory page outlining the study objectives, procedures, voluntary nature of participation, and participants’ rights. The introductory section also emphasized the anonymity and confidentiality of all collected data.

Participants were informed that no personally identifiable information would be collected and that all responses would be used solely for research purposes. Submission of the completed questionnaire was considered as provision of informed consent to participate in the study. Students were further assured that participation was entirely voluntary and that refusal or withdrawal from the study would not affect their academic standing or relationship with their universities in any way.

## Ethical consideration

Ethical approval for this study was obtained from the IRB of An-Najah National University (Approval No. Nurs.Dec.2024/12). The study was conducted in accordance with the ethical principles outlined in the Declaration of Helsinki. Electronic informed consent was obtained from all participants prior to data collection. Participants were provided with detailed information regarding the study objectives, procedures, potential risks and benefits, confidentiality measures, and their rights as research participants. Participation was entirely voluntary, and participants were informed of their right to withdraw from the study at any time without penalty or academic consequences. Completion and submission of the online questionnaire were considered indicative of informed consent. Participant anonymity and confidentiality were strictly maintained throughout the study. No personally identifiable information was collected, and all data were anonymized using unique study codes. Electronic data were securely stored on password-protected devices accessible only to the research team.

### Data analysis

Data were analyzed using IBM SPSS Statistics version 27. Descriptive statistics were used to summarize participant characteristics and study variables. Categorical variables were presented as frequencies and percentages, whereas continuous variables were summarized using means and standard deviations. Spearman’s rank correlation analysis was performed to examine the bivariate relationships between suicidal ideation scores and participants’ characteristics. Multiple linear regression analysis was then conducted to identify factors independently associated with suicidal ideation. The total BSS score was treated as the continuous dependent variable, while age, previous history of mental illness, economic status, and sleep pattern were entered as independent variables. Prior to model estimation, the assumptions of multiple linear regression were evaluated. Linearity between continuous predictors and suicidal ideation scores was assessed using scatterplots. Independence of residuals was examined using the Durbin–Watson statistic. Normality and homoscedasticity of residuals were evaluated through histogram and residual plots. Multicollinearity among predictor variables was assessed using Variance Inflation Factor (VIF) and tolerance statistics. All variables included in the final model demonstrated acceptable multicollinearity (VIF < 2.0 and tolerance > 0.50). Standardized regression coefficients (β), unstandardized coefficients (B), standard errors (SE), 95% confidence intervals (CI), and p-values were reported. Statistical significance was established at *p* < 0.05.

## Results

### Participant sociodemographic characteristics

A total of 646 nursing students participated in the study. The sociodemographic characteristics of the participants are presented in Table 1. More than half of the participants were aged between 18 and 20 years (50.9%). The majority of respondents were female (72.1%) and single (87.8%). Regarding religious affiliation, nearly all participants identified as Muslim (99.1%). In terms of nationality, most participants were from Jordan (76.8%), while 23.2% were from Palestine. Most participants reported a moderate socioeconomic status (85.8%). Participants were relatively evenly distributed across academic years, with the highest proportion enrolled in the third year (28.2%), followed by the second year (25.9%), fourth year (23.4%), and first year (22.6%). The majority of participants (86.2%) reported no previous history of mental illness, whereas 13.8% indicated a prior mental health condition. With respect to sleep patterns, approximately half of the participants reported sleeping 4–6 h per day (48.5%), while 47.7% reported sleeping more than 6 h daily. Only 3.9% of participants reported sleeping fewer than 4 h per day.

**Table 1:** Participants’ sociodemographic characteristics (*N* = 646)

Variable	Frequency (*n*)	Percentage (%)
**Age (Years)**		
18–20 21–23 ≥ 24	329 205 112	50.9 31.7 17.4
**Gender**		
Female Male	466 180	72.1 27.9
**Marital status**		
Single Married Divorced	567 73 6	87.8 11.3 0.9
**Religion**		
Muslim Other	640 6	99.1 0.9
**Nationality**		
Jordan Palestine	496 150	76.8 23.2
**Economic status**		
Low Moderate High	26 554 66	4.0 85.8 10.2
**Academic year**		
1st year 2nd year 3rd year 4th year	146 167 182 151	22.6 25.9 28.2 23.4
**Previous mental illness**		
No Yes	557 89	86.2 13.8
**Sleeping hours**		
< 4 h 4–6 h > 6 h	25 313 308	3.9 48.5 47.7

### Prevalence of suicidal ideation among nursing students

Using the recommended cutoff score of ≥ 6 on the BSS, 490 participants (75.9%) were classified as having elevated suicidal ideation, whereas 156 participants (24.1%) scored below the cutoff (Table 2**)**. These prevalence estimates were derived from the dichotomized BSS scores and were used solely to estimate the proportion of students with elevated suicidal ideation.

**Table 2:** Prevalence of suicidal ideation among nursing students* (*N* = 646)

Suicidal ideation categories	Frequency (*n*)	Percentage (%)
No suicidal ideation	156	24.1
Present suicidal ideation	490	75.9
Total	646	100

*Elevated suicidal ideation was defined as a BSS total score ≥ 6

### Correlations between participants’ characteristics and suicidal ideation scores

Table 3 presents the bivariate correlations between participants’ sociodemographic characteristics and the continuous BSS total score. A previous history of mental illness demonstrated the strongest positive correlation with suicidal ideation scores (*r* = 0.21, *p* < 0.01), indicating that students reporting a previous mental health condition tended to have higher suicidal ideation scores. Economic status also showed a weak positive correlation with suicidal ideation (*r* = 0.14, *p* < 0.01). Conversely, age was weakly and negatively correlated with suicidal ideation scores (*r* = − 0.09, *p* < 0.05), suggesting that younger students tended to report higher levels of suicidal ideation. Sleep duration demonstrated a weak negative correlation with suicidal ideation scores (*r* = − 0.08, *p* < 0.05). No statistically significant correlations were observed between suicidal ideation scores and sex, marital status, religion, nationality, or academic year.

**Table 3:** Correlation between sociodemographic variables and suicidal ideation

Variable	1	2	3	4	5	6	7	8	9	10
1. Age	1									
2. Sex	0.07	1								
3. Marital status	0.54**	−0.03	1							
4. Religion	−0.04	−0.08*	−0.01	1						
5. Nationality	−0.18**	−0.04	−0.14**	−0.10*	1					
6. Academic year	0.35**	−0.02	0.09*	−0.03	0.01	1				
7. Previous mental illness	−0.01	0.00	0.02	−0.01	−0.01	−0.05	1			
8. Economic status	0.12**	0.02	0.01	0.06	−0.02	0.01	0.09*	1		
9. Sleep pattern	−0.01	−0.08*	−0.07	−0.01	−0.02	0.06	−0.04	−0.09*	1	
10. Suicidal ideation (total score)	−0.09*	0.00	−0.05	−0.07	0.02	−0.05	0.21**	0.14**	−0.08*	1

Spearman rank correlation coefficients (r), ** Correlation is significant at *P* < 0.01

### Multiple linear regression analysis of factors associated with suicidal ideation scores

A multiple linear regression analysis was conducted to identify factors independently associated with BSS total scores. The independent variables entered into the model were age, previous history of mental illness, perceived economic status, and sleep duration. Prior to the analysis, the assumptions of multiple linear regression were assessed, and no evidence of problematic multicollinearity was identified among the independent variables.

Prior to conducting the multiple linear regression analysis, multicollinearity among the independent variables was assessed using tolerance and VIF statistics. No evidence of problematic multicollinearity was identified. Tolerance values ranged from 0.97 to 0.99, while VIF values ranged from 1.01 to 1.03, indicating that the independent variables were not highly correlated and that the assumptions for multiple linear regression were satisfied. Following confirmation that the regression assumptions were met, the overall regression model was statistically significant (F(4, 641) = 12.88, *p* < 0.001), explaining 7.4% of the variance in suicidal ideation scores (R² = 0.074; adjusted R² = 0.069). The observed regression model corresponded to a small effect size (Cohen’s f² =0.08), indicating that the included predictors explained a modest proportion of the variance in suicidal ideation scores. As shown in Table 4, younger age (B = − 0.59, β = −0.10, *p* = 0.010), a previous history of mental illness (B = 2.54, β = 0.20, *p* < 0.001), and higher perceived economic status (B = 1.50, β = 0.13, *p* = 0.001) were independently associated with higher suicidal ideation scores. Although sleep duration demonstrated a significant correlation with suicidal ideation in the bivariate analysis, it was not independently associated with suicidal ideation after adjustment for the other variables in the regression model (B = − 0.56, β = −0.07, *p* = 0.059).

**Table 4:** Multiple linear regression analysis of factors associated with BSS scores (*N* = 646)

Predictor	B	SE	β	t	*p*	95% CI for B	Tolerance	VIF
Constant	3.92	1.37	—	2.86	0.004	1.22, 6.61	—	—
Age	−0.59	0.23	−0.10	−2.60	0.010	−1.03, − 0.14	0.98	1.02
Previous mental illness	2.54	0.49	0.20	5.17	< 0.001	1.58, 3.51	0.99	1.01
Economic status	1.50	0.46	0.13	3.26	0.001	0.59, 2.40	0.97	1.03
Sleep pattern	−0.56	0.30	−0.07	−1.89	0.059	−1.15, 0.02	0.99	1.01

All VIF values were <2.0 and tolerance values >0.50

Model statistics: F(4, 641) = 12.88, p < 0.001; R² = 0.074; Adjusted R² = 0.069; Standard error of the estimate = 4.29

## Discussion

The present study examined the prevalence of suicidal ideation and its associated factors among undergraduate nursing students in Jordan and Palestine. Using the recommended cutoff score (≥ 6) on the BSS, approximately three-quarters (75.9%) of participants were classified as experiencing elevated suicidal ideation. Although this prevalence appears substantially higher than that reported in most previous studies among nursing students, which generally ranges from 13.6% to 52.5% [27–30], comparisons should be interpreted cautiously because prevalence estimates vary according to the assessment instruments, cutoff scores, study populations, and contextual circumstances. For example, the Iranian study reporting a prevalence of 70.3% [31] used a comparable BSS-based assessment; however, direct comparisons require consideration of differences in study populations and operational definitions of suicidal ideation. Moreover, the cutoff score of ≥ 6 identifies a broad spectrum of suicidal thoughts, including mild or passive suicidal ideation, rather than exclusively active suicidal planning or intent. Consequently, the prevalence observed in the present study reflects elevated suicidal ideation requiring further assessment rather than clinically confirmed suicide risk.

The high prevalence observed may also reflect the unique sociopolitical and educational context in which many participants pursue their nursing education. Nursing students in both Jordan and Palestine experience demanding academic workloads and emotionally challenging clinical training. In Palestine, these educational stressors occur within a broader environment characterized by prolonged political instability, movement restrictions, recurrent conflict, and chronic uncertainty. Recent evidence indicates that Palestinian university students exposed to repeated conflict-related stressors—including military incursions, checkpoint delays, restrictions on movement, educational disruption, and chronic insecurity—experience significantly poorer psychological well-being and quality of life, accompanied by higher levels of anxiety, depression, and psychological distress [32, 33]. These chronic environmental stressors may compound the already demanding academic and clinical requirements of nursing education, potentially contributing to the elevated levels of suicidal ideation observed in the present study.

Among the variables examined, a previous history of mental illness demonstrated the strongest independent association with higher suicidal ideation scores. This finding is consistent with previous studies reporting strong associations between suicidal ideation and pre-existing mental health conditions, particularly depression, anxiety, and psychological distress [30, 34–36]. Individuals with a history of mental illness frequently experience persistent negative cognitive patterns, emotional dysregulation, hopelessness, and maladaptive coping strategies, all of which have been associated with suicidal thinking [31, 37–39]. Within nursing education, these psychological vulnerabilities may be further challenged by intensive academic demands, repeated examinations, emotionally demanding clinical placements, and frequent exposure to patient suffering and death. Nevertheless, given the cross-sectional nature of the present study, the temporal relationship between previous mental illness and suicidal ideation cannot be established.

An unexpected finding of the present study was the positive association between higher perceived economic status and suicidal ideation. Although this finding contrasts with numerous previous studies demonstrating greater suicidal ideation among students experiencing financial hardship [40–42], similar observations have been reported in some university populations [43]. This association should therefore be interpreted cautiously. Rather than reflecting a direct effect of socioeconomic status itself, higher perceived economic status may be associated with greater academic expectations, increased parental or societal pressure to achieve, and perfectionistic tendencies, all of which have been linked to psychological distress among university students [44]. Furthermore, perceived economic status represents a subjective measure that may capture social comparison and perceived expectations rather than objective financial resources. Consequently, longitudinal studies are warranted to further explore the complex relationship between socioeconomic position and suicidal ideation in nursing students.

The present study also found that younger age was independently associated with higher suicidal ideation scores. This finding is consistent with previous studies reporting greater psychological vulnerability among younger university students [45–47]. Students in the early stages of university education often experience substantial transitions, including adjustment to new academic environments, increased independence, evolving social relationships, and uncertainty regarding their professional future. These developmental challenges may reduce resilience and increase susceptibility to psychological distress. In contrast, older students may have developed more effective coping strategies, stronger social support networks, greater emotional maturity, and increased confidence in managing academic and clinical responsibilities, which may partially explain the observed association.

Although sleep duration demonstrated a weak negative correlation with suicidal ideation in the bivariate analysis, it was not independently associated with suicidal ideation after adjustment for other variables in the multiple linear regression model. Therefore, sleep duration should not be interpreted as an independent predictor within the present study. Nevertheless, previous research has consistently reported associations between poor sleep quality, insomnia, inadequate sleep duration, and suicidal ideation among university students [48–51]. Future longitudinal studies incorporating comprehensive measures of sleep quality may provide further insight into the role of sleep disturbances in suicidal ideation among nursing students.

The findings of the present study have important implications for nursing education and student mental health services. Given the high proportion of students reporting elevated suicidal ideation, nursing faculties should consider implementing routine mental health screening, early identification of psychologically vulnerable students, and timely referral pathways for appropriate mental health care. Universities should also strengthen psychological counseling services and incorporate evidence-based programs focusing on stress management, resilience, emotional regulation, and adaptive coping skills throughout nursing education. Because nursing students are exposed to both academic and clinical stressors, interventions should be tailored to address the unique emotional demands of nursing education while remaining culturally sensitive to the sociocultural context of Jordan and Palestine.

This study has several limitations that should be acknowledged. The cross-sectional design precludes conclusions regarding causal relationships between the observed variables and suicidal ideation. Additionally, the use of a self-administered online questionnaire may have introduced reporting and response biases, while the convenience sampling approach may limit the generalizability of the findings and increase the potential for selection bias. Furthermore, suicidal ideation was classified using a screening cutoff rather than a clinical diagnostic assessment. Despite these limitations, the study provides important evidence regarding the prevalence of elevated suicidal ideation among undergraduate nursing students in two Middle Eastern countries and identifies previous mental illness, younger age, and perceived economic status as factors independently associated with higher suicidal ideation scores. These findings support the need for culturally appropriate mental health promotion strategies, routine psychological screening, and evidence-based suicide prevention initiatives within nursing education programs.

## Conclusion

This study found a high prevalence of elevated suicidal ideation among undergraduate nursing students in Jordan and Palestine. Previous history of mental illness, younger age, and higher perceived economic status were independently associated with higher suicidal ideation scores. These findings highlight the need for routine mental health screening and early identification of psychologically vulnerable nursing students, alongside strengthening accessible and culturally appropriate psychological support services within nursing education programs. Integrating evidence-based mental health promotion, resilience-building, and suicide prevention initiatives into undergraduate nursing curricula may help support students’ psychological well-being throughout their academic and clinical training. Future longitudinal and multicenter studies are warranted to further elucidate the temporal relationships among these factors and to evaluate the effectiveness of targeted interventions aimed at reducing suicidal ideation among nursing students.

## Supplementary Information

Below is the link to the electronic supplementary material.

Supplementary Material 1

## Data Availability

The datasets generated and/or analyzed during the current study are available from the corresponding author upon reasonable request and subject to ethical approval.

## Abbreviations

BSS: Beck Scale for Suicidal Ideation
IRB: Institutional Review Board
