# Supplementary material for: Prevalence and factors associated with suicidal ideation among undergraduate nursing students: a cross-sectional study
Source: BMC Nurs. 2026 Aug 18;25:803. doi: 10.1186/s12912-026-05259-7 (PMC13543389; doi:10.1186/s12912-026-05259-7)
Supplement: Supplementary file 1 [file 12912_2026_5259_MOESM1_ESM.pdf]

The First Section: Sociodemographic Data

In this section, the following social and demographic information will be collected:

Question	Response category
**What is your age?**	18-20 years<br>21-23 years<br>24 years or older
**Gender:**	Male<br>Female
**Marital status:**	Single<br>Married<br>Divorced
**Religion:**	Muslim<br>Other
**Economic status:**	Low<br>Medium<br>High
**Academic year:**	First year<br>Second year<br>Third year<br>Fourth year
**Nationality:**	Jordan<br>Palestine
**History of mental illness:** "Have you ever been diagnosed with a mental health disorder (e.g., depression, anxiety)?"	Yes<br>No
**Sleep Patterns:** "How many hours do you sleep in a day?"	Less than 4 hours<br>4-6 hours<br>More than 6 hours
